# Complete Mitochondrial Genomes of Four *Pelodiscus sinensis* Strains and Comparison with Other Trionychidae Species

**DOI:** 10.3390/biology12030406

**Published:** 2023-03-03

**Authors:** Jing Chen, Jinbiao Jiao, Xuemei Yuan, Xiaohong Huang, Lei Huang, Lingyun Lin, Wenlin Yin, Jiayun Yao, Haiqi Zhang

**Affiliations:** Agriculture Ministry Key Laboratory of Healthy Freshwater Aquaculture, Key Laboratory of Fish Health and Nutrition of Zhejiang Province, Key Laboratory of Fishery Environment and Aquatic Product Quality and Safety of Huzhou City, Zhejiang Institute of Freshwater Fisheries, Huzhou 313001, China

**Keywords:** *Pelodiscus sinensis*, mitochondrial genome, structural variation, control region, phylogenetic relationship

## Abstract

**Simple Summary:**

The Chinese soft-shelled turtle (*Pelodiscus sinensis*) is an economically important aquatic reptile species with rich nutrition and medicinal value. However, the wild resources of *P. sinensis* have been depleting due to natural and artificial factors in recent decades. Herein, we report the complete mitochondrial genomes of four *P. sinensis* strains and analyzed the nucleotide composition and variable site for the four mitogenomes. Using Ka/Ks and sliding window analyses, we explored the genetic diversity and selection pressures of different mitochondrial genes in the four *P. sinensis* strains. Through comparative analysis, the present study described the structural variation of 22 tRNAs, replication origin region of the L-strand, and control region, indicating the genetic variations among the four *P. sinensis* strains. Furthermore, the evolutionary relationship of *P. sinensis* strains and other Trionychidae species was determined by phylogenetic analysis. Taken together, our findings provide genetic information and an essential basis for understanding the genetic variations and evolutionary relationship of *P. sinensis* strains, which will play an important role in bioprospecting and conservation of *P. sinensis*.

**Abstract:**

The Chinese soft-shelled turtle (*Pelodiscus sinensis*) is an important aquaculture reptile with rich nutritional and medicinal values. In recent decades, the wild resources of *P. sinensis* have been depleting due to natural and artificial factors. Herein, we report the complete mitochondrial genome of four *P. sinensis* strains, including the Japanese (RB) strain, Qingxi Huabie (HB) strain, Jiangxi (JB) strain, and Qingxi Wubie (WB) strain. The nucleotide composition within the complete mitogenomes was biased towards A + T with a variable frequency ranging from 59.28% (*cox3*) to 70.31% (*atp8*). The mitogenomes of all four strains contained 13 protein-coding genes (PCGs), 22 tRNAs, 2 rRNAs, 1 control region, and a replication origin region of the L-strand replication (OL), which was consistent with most vertebrates. Additionally, the *atp8*, *nad4l*, *nad6*, and *nad3* genes possessed high genetic variation and can be used as potential markers for the identification of these *P. sinensis* strains. Additionally, all PCGs genes were evolving primarily under purifying selection. Through comparative analysis, it was revealed that most of the tRNAs were structurally different in the TψC stem, DHU stem, and acceptor stem. The length of the tandem repeats in the control region was variable in the four *P. sinensis* strains, ranging from 2 bp to 50 bp. Phylogenetic analysis indicated that all *P. sinensis* strains clustered into one branch and were closely related to other Trionychinae species. Overall, this study provides mitochondrial genome information for different *P. sinensis* strains to support further species identification and germplasm resource conservation.

## 1. Introduction

The Chinese soft-shelled turtle (*Pelodiscus sinensis*) belongs to the genus *Pelodiscus* of the family Trionychidae, and it is recognized as an important economic species with nutritional value and medicinal value [1,2,3]. *P. sinensis* is widely distributed in East Asia, including China, Korea, Japan, and Vietnam [4,5]. According to the geographical distribution, *P. sinensis* can be divided into different strains, including the Japanese strain, Yellow River strain, Huaihe River strain, Poyang Lake strain, Taihu Lake strain, Southwest strain, Taiwan strain, etc. [6]. In recent years, two new strains of *P. sinensis* (Qingxi Huabie and Wubie strains) have been identified, which originated from Taihu Lake basin and are unique local strains in Zhejiang Province [7]. Different *P. sinensis* strains have their own traits, such as fast growth rate for the Japanese strain and light-yellow body color for the Yellow River strain [6]. With the continuous improvement in life quality, the consumption demand for *P. sinensis* has also been increasing. The state of world fisheries and aquaculture in 2022 reported that the annual output of *P. sinensis* was 334.3 thousand tons in 2020, accounting for 31.5% of aquatic animals (except finfish, crustaceans, and mollusks) [8]. In the past decades, the scale of artificial breeding of *P. sinensis* has been expanding, and many breeding individuals in non-original wild habitats have escaped to natural waters, causing serious damage to wild germplasm resources [9]. Meanwhile, due to over-fishing and the destruction of the natural environment, the wild *P. sinensis* population has decreased sharply and almost dried up [10,11]. The *P. sinensis* has been listed in the United Nations Red Book on Endangered Species (http://www.iucnredlist.org/details/39620/0) (accessed on 20 October 2022). Until now, the classification of different *P. sinensis* strains has mainly depended on morphological traits, and only a few studies have involved molecular systematics, which plays a crucial role in germplasm resource conservation of *P. sinensis*.

To date, many methods have been developed for species classification and phylogenetic research based on DNA sequencing [12,13,14]. Among them, mitochondrial DNA (mtDNA) is widely used for its unique advantages; it is rarely affected by gene recombination, it strictly has maternal inheritance in vertebrates, and it has a high mutation rate [15,16,17]. The rapid evolution of mtDNA implies that many sequence variations can be identified among closely related species, which is an effective tool for species identification [18]. For most vertebrates, the mitochondrial genome contains thirteen protein-coding genes (seven subunits of complex I, one subunit of complex III, three subunits of complex IV, and two subunits of complex V); two rRNAs (12S rRNA and 16S rRNA); one control region (D-loop); and twenty-two tRNAs [19,20]. At present, many studies have reported the phylogenetic relationship and species identification of Trionychidae species by mtDNA analysis. For instance, Zhang et al. reported that *Trionyx sinensis* had a close relationship with *P*. *axenaria* as determined by the mitochondrial *cytb* gene [21]. Liang et al. amplified the mitochondrial *cox1* gene and found that the Huaihe River strain of *P. sinensis* was more closely related to the Yellow River strain than the Japanese strain [22]. Using the mitochondrial D-loop, Li et al. demonstrated that the Tai Lake population and Hongze Lake population of *P. sinensis* had a close genetic relationship [23]. Chen et al. used the mitochondrial 12S rRNA to identify *P. axenaria* as a new species [24]. Zhang et al. sequenced and analyzed the partial sequences of *nad4*, *cox1*, *nad5*, and *nad6* genes and further confirmed the identification of *P. sinensis* strains [6]. However, most studies only involved some gene fragments of the mitochondrial genome. Increasing evidence has revealed that the complete mitochondrial genome could provide accurate insight into genetic differentiation and species identification [25,26].

In the present study, we amplified and analyzed the complete mitochondrial genome of four *P. sinensis* strains, including the Japanese strain, Jiangxi strain, Qingxi Huabie strain, and Qingxi Wubie strain. To investigate the mitochondrial genome features and genetic variations of the four *P. sinensis* strains, the present study was designed as follows: (1) compare and analyze the length, nucleotide composition, and variable sites of the four mitogenomes; (2) explore the genetic diversity and selection pressures of different mitochondrial genes in the four *P. sinensis* strains; (3) illustrate the structural variation of 22 tRNAs, the origin of L-strand replication, and the control region among the four *P. sinensis* strains; (4) determine the phylogenetic relationship of *P. sinensis* strains and other Trionychidae species. These results offer abundant genetic information for the four *P. sinensis* strains and will be conducive to the conservation and utilization of germplasm resources of *P. sinensis*.

## 2. Materials and Methods

### 2.1. Sample Information

In this study, healthy *P. sinensis* samples with about 150 g weight, including the Japanese (RB) strain, Jiangxi (JB) strain, Qingxi Wubie (WB) strain, and Qingxi Huabie (HB) strain, were collected from a breeding base of Zhejiang province. The liver tissue of these strains was collected and stored at −80 °C. The study was approved by the Institutional Animal Care and Use Committee (IACUC) of the Zhejiang Institute of Freshwater Fisheries.

### 2.2. DNA Extraction, Sequencing, and Assembly

Genomic DNA was extracted using a genomic DNA extraction kit (Tiangen, Beijing, China). The quantity and quality of DNA was detected by NanoDrop 2000 (Thermo Fisher Scientific, Waltham, MA, USA) and agarose gel electrophoresis. According to the published mitochondrial genome sequence of *P. sinensis* in GenBank (https://www.ncbi.nlm.nih.gov/nuccore/AY687385.1) (accessed on 12 June 2022), the primers used to amplify the complete mitochondrial genome of these strains were designed by Primer premier 5.0 software (Table 1) [27]. The amplification reactions were performed in a total volume of 50 µL, including 19 µL of ddH_2_O, 25 µL of 2 × Taq Master Mix (Takara, Shiga, Japan), 2 µL of each primer (10 µM/L), and 2 µL of genomic DNA (120 ng/µL). The PCR cycle was an initial denaturation at 95 °C for 3 min; 35 cycles of 95 °C for 30 s, 58 °C for 30 s, and 72 °C for 1 min/Kb (1 min per Kb amplification length); and a final extension at 72 °C for 5 min. PCR amplification products were detected by agarose gel electrophoresis. After purification, the PCR product was sequenced by Sanger sequencing in Tsingke Biological Technology (Beijing, China). The regions containing prominent repeats were amplified into different fragments and then sequenced using the primer walking method. Cluster X 2.0 software [28] and BioEdit 7.0 software [29] were used to align and correct the obtained sequences, and the complete mitochondrial genome sequence was obtained by SeqMan software (DNAStar Inc., Madison, WI, USA) [30].

### 2.3. Mitogenome Annotation and Sequence Analysis

The sequence annotation was conducted in the ARWEN and MITOS (http://mitos.bioinf.uni-leipzig.de) (accessed on 15 September 2022) online servers [31,32]. The initiation and termination codons of protein-coding genes (PCGs) were identified using other reference sequences of Trionychidae species. The online program OGDraw v1.2 with default parameters was used to map the circular map of the completed mitochondrial genome [33]. Subsequently, the base composition and codon usage of the mitochondrial genome of four *P. sinensis* strains were analyzed by MEGA X software [34], and the AT skew = (A − T)/(A + T) and GC skew = (G − C)/(G + C) were calculated by the method previously reported [35]. Relative synonymous codon usage (RSCU) is an important indicator to judge codon usage preference. The RSCU value represents the ratio of the usage bias of a codon to its expected usage bias in the synonymous codon family (all codons for a particular amino acid are used equally). Codons with an RSCU value > 1.0 have positive codon usage bias, while codons with an RSCU value < 1.0 have negative codon usage bias [36]. The software MEGA X was used to calculate RSCU values. Furthermore, multiple sequence alignment for the mitochondrial genomes of four *P. sinensis* strains was performed using MEGA X software. Then, the PCGs, rRNAs, tRNAs, and control region (D-loop) of four *P. sinensis* strains were analyzed by DnaSPv6.0 software for gene traits and variation sites [37]. The software DnaSPv6.0 was used to calculate the synonymous substitutions per synonymous sites (Ks) and non-synonymous substitutions per non-synonymous sites (Ka). The Ka/Ks ratio was used to assess the selection pressure, Ka/Ks > 1 indicated a positive selection, Ka/Ks = 1 indicated a neutral selection, and Ka/Ks < 1 indicated a purifying (stabilizing) selection [38]. The genetic distance was analyzed by MEGA X software. In addition, the nucleotide diversity (Pi) of 13 PCGs and 2 rRNAs were analyzed by sliding window analysis (500 bp windows every 10 bp) using DnaSPv6.0 software.

### 2.4. Structural Analyses of Mitogenome and Prediction of Repeat Element

To determine the unique base compositions in the control regions (CRs) of four *P. sinensis* strains, tandem repeats were predicted by the online Tandem Repeats Finder web tool (https://tandem.bu.edu/trf/trf.html) (accessed on 17 October 2022) [39]. For the four *P. sinensis* strains, the stem-loop structures of the origin of L-strand replication were analyzed by the online Mfold web server (http://www.unafold.org/) (accessed on 17 October 2022) [40]. The 22 tRNAs of mitogenomes of the four *P. sinensis* strains were verified in MITOS online server (http://mitos.bioinf.uni-leipzig.de) (accessed on 18 October 2022). Then, the online tRNAscan SE Search Server 2.0 (http://lowelab.ucsc.edu/tRNAscan-SE/) (accessed on 19 October 2022) and RNAstructure software were used to predict the variation in tRNA secondary structure among the mitogenomes of four *P. sinensis* strains [41,42], and their mutation sites were analyzed. The base composition of all components (DHU arm, acceptor stem, TψC arm, an anti-codon arm) were manually checked to distinguish the mutation sites.

### 2.5. Construction of Phylogenetic Tree

Phylogenetic analysis was performed on the dataset of 13 PCGs, 22 tRNAs, and 2 rRNAs from 19 Trionychidae mitogenomes published in GenBank and four *P. sinensis* mitogenomes sequenced in this study. Sequence alignment of 13 PCGs was performed using MAFFT with default settings in PhyloSuite v1.2.3 [43,44], and the aligned sequences were checked using MEGA X. The best-fit partitioning scheme and corresponding nucleotide substitution models for concatenated nucleotides were selected using PartitionFinder v2.1.1 according to the Bayesian information criterion (BIC) (Table S1) [45]. Phylogenetic trees of concatenated nucleotide sequences were constructed with the best-fit partitioning schemes and nucleotide substitution models using Bayesian inference (BI) and maximum-likelihood (ML) methods in MrBayes v3.2.1 and IQ-tree v2.0.4, respectively [46,47]. Using the best-fit model, the ML analysis was run for each partition with 2000 ultrafast bootstrap (UFB) replicates and performed until a correlation coefficient of at least 0.99 was reached [48,49]. In addition, the BI analysis was run independently using four Markov Chain Monte Carlo (MCMC) chains (three heated chains and one cold chain) starting with a random tree; each chain was run for 2 × 10^7^ generations and sampled every 1000 generations. Convergence of data runs was estimated by the average standard deviation of split frequencies (ASDSF) < 0.01. The phylogenetic trees were visualized in FigTree v1.4.4 [50].

## 3. Results and Discussion

### 3.1. Analysis of Mitogenome Features

**Figure 1:**
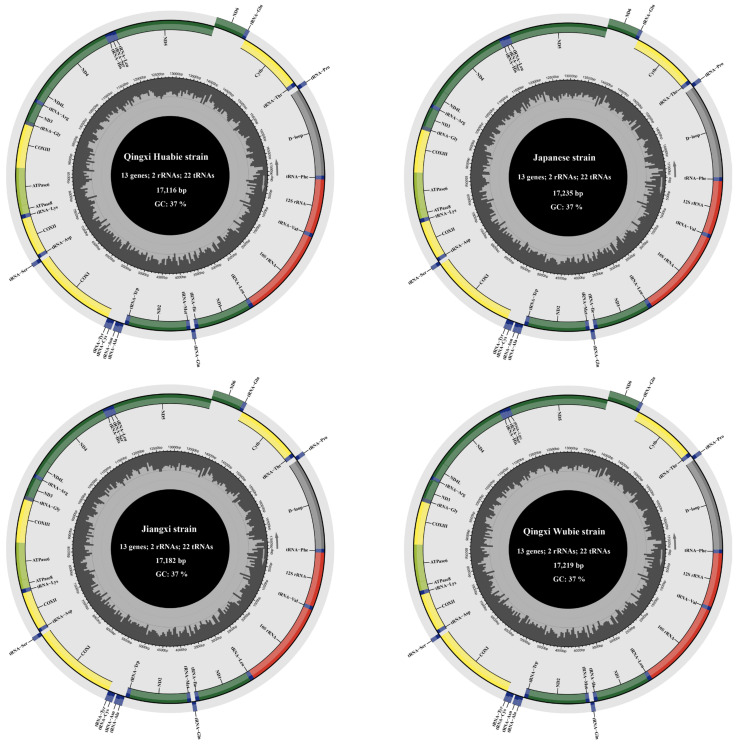
**Mitochondrial genome maps of four *P. sinensis* strains**. The outer circle represents the light chain (L-chain), and the inner circle represents the heavy chain (H-chain). The dark green box represents mitochondrial complex I (NADH dehydrogenase); the yellow box represents complex IV (cytochrome c oxidase); the light green circle represents ATP synthase; the blue circle represents transfer RNA; the red circle represents ribosomal RNA; and the grey circle represents the control region (D-loop).

In this study, we amplified the complete mitochondrial genomes of four *P. sinensis* strains. The result showed that the four mitogenomes were circular molecules with lengths of 17,219 bp, 17,116 bp, 17,235 bp, and 17,182 bp in WB, HB, RB, and JB strains, respectively. These mitogenomes were deposited in GenBank under the accession number OQ236104 for the RB strain, OQ236105 for the HB strain, OQ236106 for the JB strain, and OQ236107 for the WB strain. The mitogenomes of all four strains consisted of 13 protein-coding genes (PCGs), 22 tRNAs, 2 rRNAs, 1 control region, and a replication origin region of the light chain (OL). The arrangement and orientation of these genes were similar to most vertebrates [51,52,53]. Previous studies had described the length and composition of mitogenomes for other *P. sinensis* strains; for instance, the inked turtle strain of *P. sinensis* was 17,145 bp in length [9], and the Korean soft-shelled turtle was 17,042 bp in length [54]. The mitogenomes of these *P. sinensis* strains had slight differences in length and possessed the same gene arrangement and orientation. However, it was reported that the mitogenome of Mediterranean tortoises contained 23 tRNAs and 2 control regions, and the mitochondrial genes of most Testudoformes species presented a similar arrangement with vertebrates, except *Platysternon megacephalum* and *Malacochersus tornieri* [55,56]. In the mitogenomes of the four *P. sinensis* strains, only the *nad6* gene and eight tRNAs (tRNA-Glu, tRNA-Pro, tRNA-Gln, tRNA-Ala, tRNA-Asn, tRNA-Cys, tRNA-Tyr, and tRNA-Ser) were encoded on the light chain (L-chain), and all other genes were encoded on the heavy chain (H-chain) (Figure 1). Additionally, it was found that there was a slight difference in the length of PCGs, tRNAs, and rRNAs among the four *P. sinensis* strains. The majority of PCGs were initiated by an ATG start codon, with the exception of *cox1*, which was initiated by GTG. Five stop codons (TAG, AGA, TAA, AGG, and T) were found in the mitochondrial genome of four *P. sinensis* strains (Table 2). Among them, the termination codon was TAG for *nad1* and *nad2*; the termination codon was TAA for *cox2*, *atp8*, *atp6*, *nad4l*, *nad5*, and *cytb*; the termination codon was a single base (T) for *cox3* and *nad4*; and the termination codon was AGA and AGG for *cox1* and *nad6*, respectively. A previous study showed that in the mitogenomes of many animals, AGA and AGG are not used as codons of arginine but are instead used as termination codons [57]. Additionally, for the *nad3* gene, the termination codon TAG was found in the HB and WB strains, and the termination codon T was observed in the JB and RB strains.

### 3.2. Nucleotide Composition and Variation Detection

**Figure 2:**
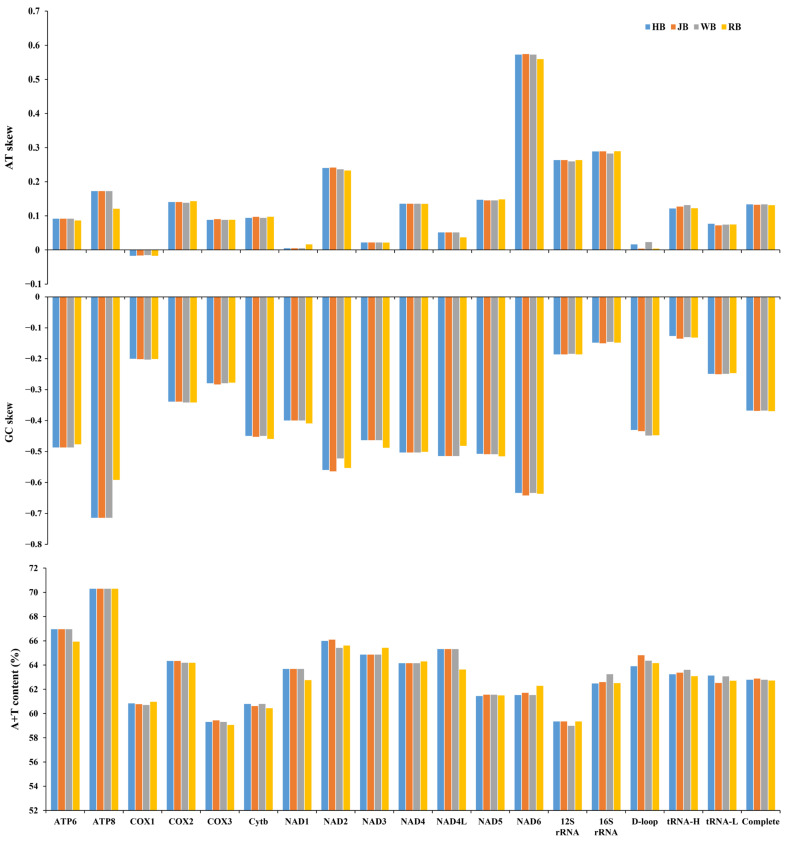
**Nucleotide compositions of mitochondrial genome elements of four *P. sinensis* strains**.

To investigate the nucleotide composition of mitogenomes, we calculated the parameters A + T content, AT-skew, and GC-skew for the four *P. sinensis* strains. The result showed that the average A + T composition was 63.74%, 63.11%, 61.40%, and 64.32% in PCGs, tRNAs, rRNAs, and D-loop, respectively (Table 3). The nucleotide composition within the complete mitogenomes was biased towards A + T with a variable frequency ranging from 59.28% (*cox3*) to 70.31% (*atp8*), which was similar to other Trionychidae species [53]. Previous studies have reported that the mitogenome of the Korean soft-shelled turtle contained 62.6% A + T content and had high nucleotide similarity with the Chinese soft-shelled turtle, and the base composition of the inked turtle strain of *P. sinensis* was 35.5% A, 27.3% T, 11.8% G, and 25.4% C, with an A + T content of 62.8% [9,54]. It was demonstrated that the mitogenomes of these *P. sinensis* strains had a similar A + T content. The average AT- and GC-skew of the complete mitogenome was 0.13 and −0.37, respectively. The AT-skew of all genes varied from −0.02 (*cox1*) to 0.57 (*nad6*), and the GC-skew ranged from −0.68 (*atp8*) to −0.16 (rRNAs) (Table 3). The AT- and GC-skew in most genes indicated that more adenine (A)s/cytosine (C)s than thymine (T)s/guanine (G)s existed in the complete mitogenomes, suggesting no obvious difference among the four *P. sinensis* strains (Figure 2).

**Figure 3:**
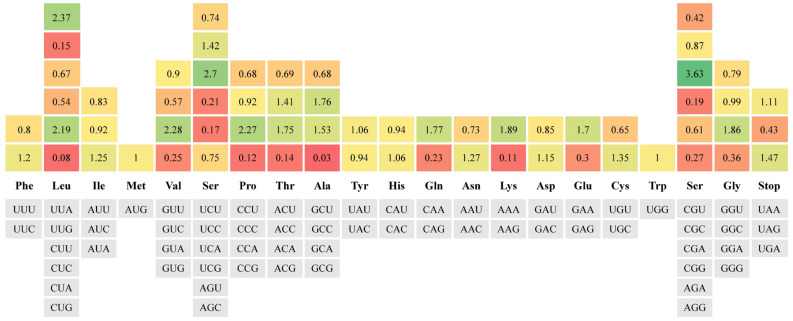
**The RSCU values of all protein-coding genes for four *P. sinensis* strains**. The red means a low RSCU value, the green indicates a high RSCU value, and the yellow indicates a middle RSCU value; the darker color indicates the largest RSCU value.

Additionally, the 13 PCGs of the HB strain (11,352 bp length), JB strain (11,350 bp length), RB strain (11,350 bp length), and WB strain (11,352 bp length) were used to estimate the RSCU values. The result showed that these encoded protein sequences contained 21 amino acids; a total of 64 codons were used in the four strains (Figure 3). It was demonstrated that a total of 27 RSCU values computed in this study exceeded 1, indicating that these codons had a high-frequency usage, and most of preference codons ended in purine (A/U) due to the abundance of A/T in the mitogenomes of the four *P. sinensis* strains. Furthermore, we detected and compared the variable sites, singleton variable sites, and parsimony informative sites among the four *P. sinensis* strains. The result showed that a total of 483 variable sites were identified, accounting for 2.83% of the total sites, including 436 singleton variable sites and 47 parsimony informative sites. Moreover, there were 25 variable sites in 22 tRNAs, 113 variable sites in the D-loop, and 291 variable sites in 13 PCGs (Table 4). A previous study reported that the control region is a non-coding sequence in mtDNA, it possesses relatively low selection pressure during evolution, and it has greater polymorphism than other mitochondrial elements [58]. Among all mitochondrial elements, the D-loop, *nad3*, *nad4l*, *nad6*, and *atp8* contained a higher percentage of variable sites than other elements. Therefore, it is inferred that these highly variable elements will play an important role in the classification and identification of the four *P. sinensis* strains.

### 3.3. Nucleotide Diversity and Selection Pressures

**Figure 4:**
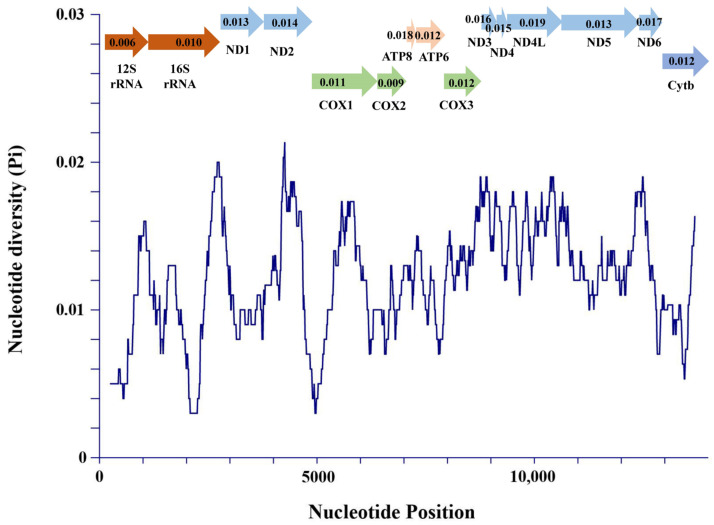
**Sliding window analysis of 2 rRNA genes and 13 PCGs among the four *P. sinensis* strains**.

**Figure 5:**
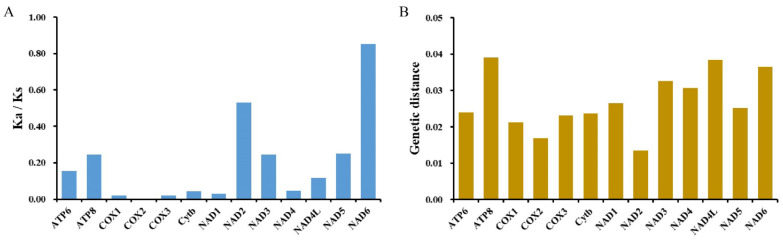
**Ka/Ks** (**A**) **and genetic distance** (**B**) **analyses of 13 PCGs among four *P. sinensis* strains**. Genetic distance indicates the overall mean distances of 13 PCGs among the four strains using the Kimura 2-parameter model of MEGA X software.

The nucleotide diversity of two rRNAs and 13 PCGs was explored using the sliding window analysis. The result showed that the nucleotide diversity levels of these genes were different. The *nad4l* gene (Pi = 0.019) had the highest nucleotide diversity compared with other PCGs genes, followed by *atp8* (Pi = 0.018), *nad6* (Pi = 0.017), and *nad3* (Pi = 0.016). Moreover, *cox2* (Pi = 0.009), *cox1* (Pi = 0.011), *cox3* (Pi = 0.012), and *cytb* (Pi = 0.012) were the most conserved genes across the PCGs and exhibited low nucleotide diversity levels (Figure 4). Compared with many other PCGs, 12S rRNA (Pi = 0.006) and 16S rRNA (Pi = 0.010) had low-level nucleotide diversity with reduced variability. To investigate the evolutionary selection constraints of the four *P. sinensis* strains, we performed Ka/Ks analysis for the 13 PCGs of mitochondrial genomes. The result showed that the Ka/Ks ratios for all PCGs were less than 1, indicating that these genes were evolving primarily under purifying selection. Among them, the lowest Ka/Ks value (0.000) for the *cox2* gene indicates the strongest purifying selection (neutral selection), whereas the highest Ka/Ks value (0.854) for the *nad6* gene showed a highly relaxed purifying selection (Figure 5A). Therefore, the Ka/Ks analysis indicated that the evolution of the four *P. sinensis* strains’ mitogenomes has been dominated by purifying selection. Similarly, Kundu et al. reported that most of the PCGs in the mitogenomes of 13 Trionychidae species showed Ka/Ks values of <1, indicating a strong purifying selection among these Trionychidae species [53]. It has been reported that advantageous alleles can be retained by either positive selection or balancing selection, while deleterious alleles are removed through purifying selection [59]. Therefore, it is inferred that purifying selection has played an important role in the elimination of deleterious mutations in the four *P. sinensis* strains during evolution. These findings provide new insights for understanding the natural selection that influences the evolution of Trionychidae species. Previous studies have revealed that purifying selection reduces genetic diversity, and the population differentiation value generated by the gene locus with strong purifying selection is lower than that of the gene locus with a fast evolution rate [60,61]. We speculate that with the continuous progress of technology, excellent varieties of *P. sinensis* have been widely promoted in recent years, and there has been a serious trend of variety simplification, which has resulted in the reduction of genetic diversity.

Furthermore, we investigated the genetic variation of 13 PCGs for four *P. sinensis* strains using genetic distance analysis. The result showed that *nad2* (average 0.014) and *cox2* (average 0.017) had low genetic distance, while *atp8* (average 0.039) possessed the largest genetic distance value, followed by *nad4l* (average 0.038), *nad6* (average 0.037), and *nad3* (average 0.033) (Figure 5B), which were the most variable genes. Taken together, the above findings reveal that the *nad3*, *nad4l*, *nad6*, and *atp8* genes possess high genetic variation and can be used as potential markers for the identification of these *P. sinensis* strains.

### 3.4. Comparative Analysis of tRNA Secondary Structure

**Figure 6:**
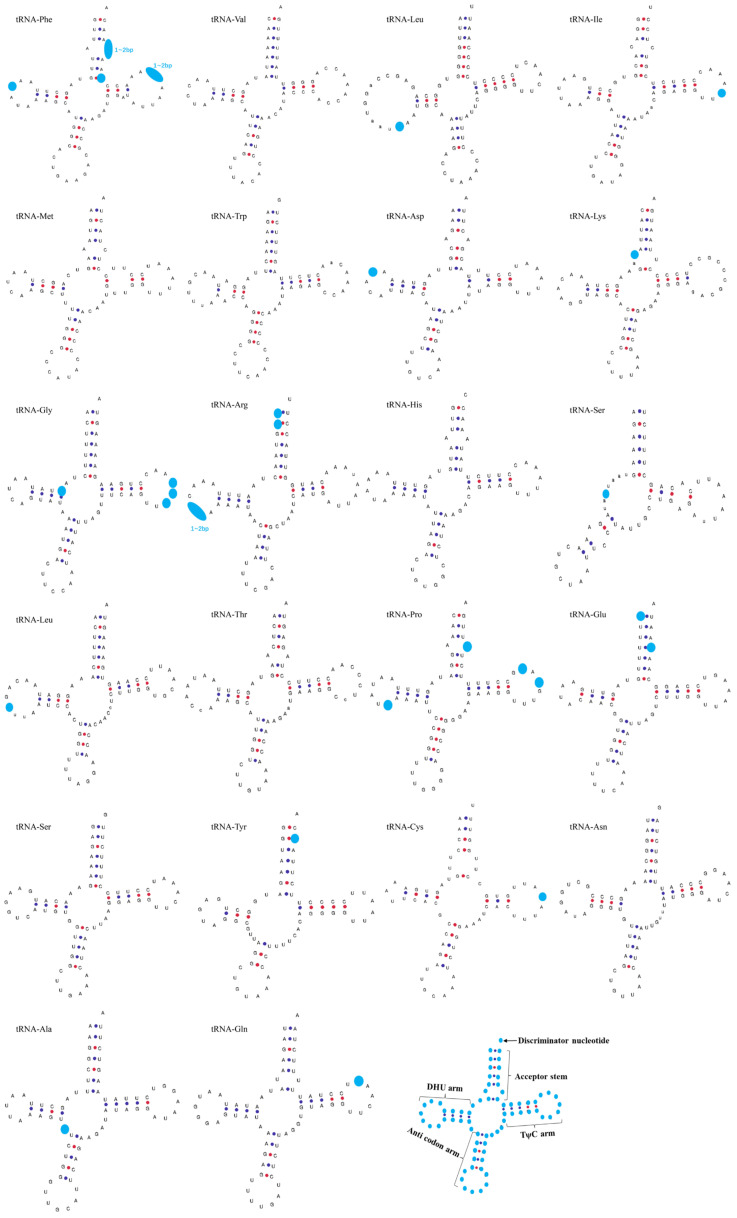
**Secondary structures of 22 transfer RNAs (tRNAs) indicating the structural variation among the four *P. sinensis* strains**. The light blue dots indicate variation sites in the tRNA secondary structure.

Extensive comparison of tRNAs is crucial for understanding the structural and functional features of the mitogenomes. Herein, we compared and analyzed the tRNA secondary structure of four *P. sinensis* strains. The result revealed that most of the tRNAs were folded into classic clover-leaf secondary structures; only the DHU stem of tRNA-Ser was missing. Interestingly, similar characteristics were also found in many other Trionychidae species [53]. Through comparative analysis, the result showed that the most variable base pairing was observed in tRNA-Phe, tRNA-Gly, tRNA-Arg, and tRNA-Pro, while unchanged base pairing was detected in tRNA-Val, tRNA-Met, tRNA-Trp, tRNA-His, tRNA-Thr, tRNA-Ser, and tRNA-Asn, which presented a similar secondary structure in the four strains (Figure 6). Additionally, it was found that most tRNAs were structurally different in the TψC stem, DHU stem, and acceptor stem. Variation in the TψC stem occurred in tRNA-Phe, tRNA-Ile, tRNA-Gly, tRNA-Pro, tRNA-Cys, and tRNA-Gln. Variation in the TψC stem and the DHU stem coexisted in tRNA-Phe and tRNA-Pro. Variation in the DHU stem was observed in tRNA-Phe, tRNA-Leu, tRNA-Asp, tRNA-Arg, tRNA-Leu, and tRNA-Pro. Furthermore, structural variation in the acceptor stem was found in tRNA-Phe, tRNA-Lys, tRNA-Arg, tRNA-Pro, tRNA-Glu, and tRNA-Tyr. Similarly, a previous study compared the structural variation of tRNAs among 13 Trionychidae species and found that most tRNAs were different in the structure of the stem and loop [53]. Moreover, it was demonstrated that the variations of 22 tRNAs were mainly base substitutions among the four *P. sinensis* strains, and a few variations involved the change in base number.

### 3.5. Comparative Analysis of Control Region

In this study, we found the origin of L-strand replication in the mitochondrial genome of four *P. sinensis* strains and detected the conserved sequences of 5′-AAAAT and AACCA-3′ in all four strains. Moreover, a stable stem-loop structure was observed in HB, JB, and WB strains with a length of 11 bp. However, due to the substitution of an adenosine with a thymidine in the RB strain, the stem-loop structure of the RB strain was only 9 bp. A previous study reported the stem-loop structure of the origin of L-strand replication in Cryptodira species but not in *Pelomedusa subrufa* of the suborder Pleurodira [62]. We speculate that this characteristic can be employed to distinguish Cryptodira and Pleurodira species, but further evidence and large-scale investigations are required for the Testudoformes species.

**Figure 7:**
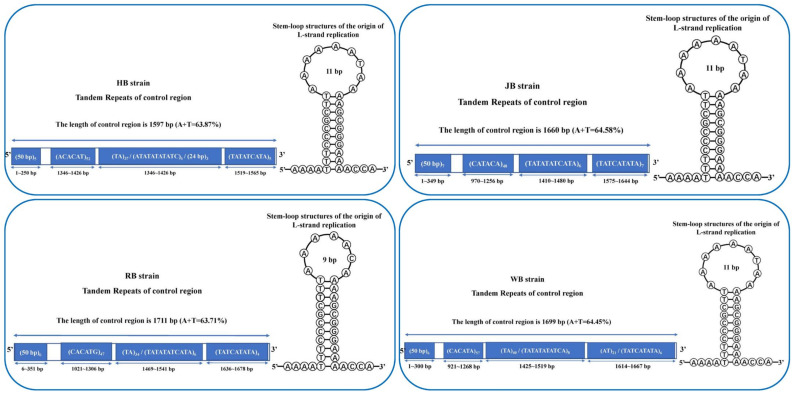
**Comparison of length, nucleotide composition in control regions (CRs), and stem-loop structures of origin of L-strand replication for four *P. sinensis* strains**.

The control region of the mitochondrial genome contains tandem repeats [63]. Herein, we observed that the length of the tandem repeats in the control region was variable in the four *P. sinensis* strains and ranged from 2 bp to 50 bp. It was demonstrated that all four *P. sinensis* strains contained four variable number tandem repeats (VNTRs); the first tandem repeat (50 bp) was found in the four strains. Moreover, the second tandem repeat of HB, JB, RB, and WB strains was (ACACAT)_52_, (CATACA)_48_, (CACATG)_47_, and (CACATA)_57_, respectively. Furthermore, the four strains exhibited different AT contents and CR length, and HB, RB, and WB strains had variable third or fourth tandem repeats. For the HB strain, the length of the CR was 1596 bp, the A + T content was 63.87%, and the third tandem repeat was (TA)_37_/(ATATATATATC)_6_/(24bp)_3_. The CR length of the RB strain was 1710 bp, the A + T content was 63.71%, and the third tandem repeat was (TA)_34_/(TATATATCATA)_6_. The CR length of the WB strain was 1698 bp, the A + T content was 64.45%, and the third and fourth tandem repeats were (TA)_40_/(TATATATATCA)_8_ and (AT)_21_/(TATCATATA)_6_, respectively (Figure 7). Unlike other strains, the CR of the JB strain contained four single tandem repeats with 1659 bp length and 64.58% A + T content. The CR length of vertebrates varies greatly, and it was suggested that the length difference of the mitochondrial genome may be mainly attributed to the change in CR length [64]. Moreover, the third and fourth tandem repeats of the JB strain were (TATATATCATA)_6_ and (TATCATATA)_7_, respectively. Our findings demonstrated that the frequency of tandem repeats was higher at the 3′ end of the CR for all four *P. sinensis* strains, and a single short tandem repeat (TA) was found in HB, RB, and WB strains. It has been reported that the CR of the mitochondrial genome is the most variable fragment as a result of the lack of coding restrictions in most species [65].

### 3.6. Phylogenetic Analyses

**Figure 8:**
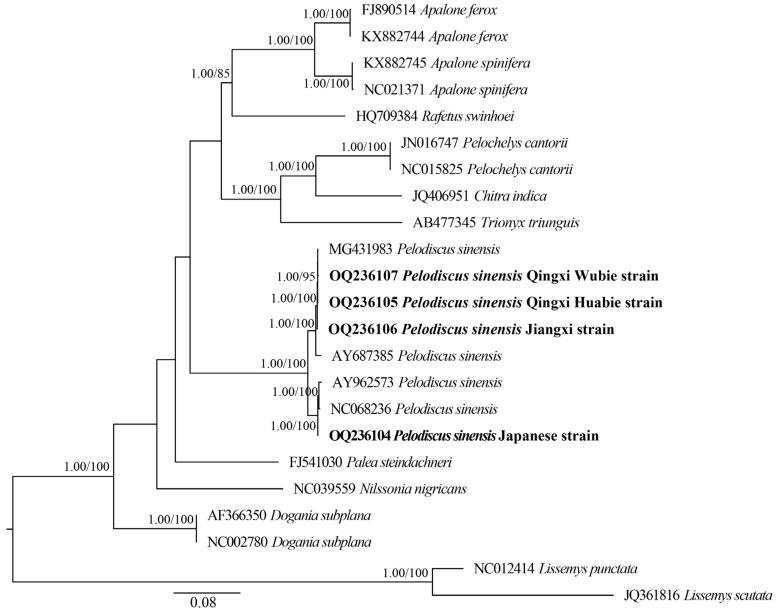
**Molecular phylogenetic analysis of Trionychidae species using BI and ML methods based on concatenated mtDNA datasets**. The phylogenetic tree nodes were considered well supported when the Bayesian posterior probability (BPP) of the node was ≥0.95 and ultrafast bootstrap was ≥80%. Numbers beside the nodes represent BPP (left) and bootstrap values (right). The bold fonts indicate the four *P. sinensis* strains sequenced in this study.

To investigate the phylogenetic relationships of *P. sinensis* strains and other Trionychidae species, we conducted a phylogenetic analysis for Trionychidae species, including the subfamily Trionychinae (*P. sinensis*, *Palea steindachneri*, *Apalone ferox*, *A. spinifera*, *Rafetus swinhoei*, *Trionyx triunguis*, *Pelochelys cantorii*, *Chitra indica*, *Dogania subplana*, and *Nilssonia nigricans*) and the subfamily Cyclanorbinae (*Lissemys punctata* and *L. scutata*). We performed the phylogenetic analysis with concatenated nucleotides (13 PCGs, 22 tRNAs, and 2 rRNAs) of 23 mitogenomes by BI and ML methods, and the phylogenetic trees constructed by these two methods had almost identical topologies. For the four strains sequenced in this study, we found that the HB and WB strains had the closest genetic relationship, with the JB strain being more closely related to them and the RB strain being farther related to other three strains (Figure 8). The HB and WB strains both originate from the Taihu Lake basin, and it is inferred that they may possess small genetic differences. Overall, the *P. sinensis* strains divided into two main branches: one containing the HB strain, WB strain, JB strain, inked turtle strain (MG431983), and *P. sinensis* from Anhui Province (AY687385) and the other containing the RB strain, the Korean soft-shelled turtle (AY962573), and *P. sinensis* from Shanghai (NC068236). It was revealed that the inked turtle strain and WB strain had a close genetic affinity with the HB strain, while the RB strain, the Korean soft-shelled turtle (AY962573), and *P. sinensis* from Shanghai (NC068236) showed a distant relationship with other *P. sinensis* strains. Using a partial 12S rRNA sequence, Xu et al. reported that the WB strain and Anhui *P. sinensis* strains clustered into one branch and then clustered with the Korean soft-shelled turtle [66]. Therefore, our findings further clarified the phylogenetic relationship of different *P. sinensis* strains and provided molecular evidence for the germplasm resource conservation of *P. sinensis*.

Additionally, the phylogenetic trees showed that all Trionychinae species were clustered together and were retrieved as a sister clade of Cyclanorbinae species, which supported the previous phylogeny of Trionychidae species [67,68]. Moreover, we observed that the species of the same genus gathered into one branch. For instance, all *Apalone* species clustered into one branch, showing a close phylogenetic relationship with *R. swinhoei*. In addition, *D. subplana* and other Trionychinae species had a distant phylogenetic relationship. However, Kundu et al. reported that *T. triunguis*, *P. cantorii,* and *C. indica* were clustered into one branch and had a distant relationship with other Trionychinae species [53]. Consequently, more sequencing data from different taxonomic ranks of Trionychidae are essential to better understand the phylogenetic and evolutionary relationships among Trionychidae species.

## 4. Conclusions

The present study described the complete mitochondrial genomes of four *P. sinensis* strains, including RB, HB, JB, and WB strains. Through comparative analysis, the following conclusions can be drawn: (1) the mitochondrial genomes of the four *P. sinensis* strains consisted of 13 PCGs, 22 tRNAs, 2 rRNAs, 1 control region, and a replication origin region of L-strand replication (OL); (2) most preference codons ended in purine (A/U) due to the abundance of A/T, and the evolution of the four *P. sinensis* strains’ mitogenomes has been dominated by purifying selection; (3) the *atp8*, *nad4l*, *nad6*, and *nad3* genes can be used as the potential markers for the identification of these *P. sinensis* strains; (4) most of the tRNAs were folded into classic clover-leaf secondary structures (except for tRNA-Ser), and the structural variation mainly involved the TψC stem, DHU stem, and acceptor stem; (5) the stem-loop structure of the OL in the RB strain was different from the other three strains, and the length of the tandem repeats in the control region was variable in the four *P. sinensis* strains and ranged from 2 bp to 50 bp; and (6) the present study further confirmed phylogenetic relationships of *P. sinensis* strains and other Trionychidae species. Therefore, our findings provide insights for the classification and evolutionary research of different *P. sinensis* strains and offer valuable genetic information for the germplasm resource conservation of Trionychidae species.

## Figures and Tables

**Table 1:** Primers used for amplification of mitochondrial genome.

Forward	Primer Sequence (5′ to 3′)	Reverse	Primer Sequence (5′ to 3′)	Product Length
Mt-1F	AGTGAAAATGCCCTAAAAGTCACATC	Mt-1R	ATACTTATTGTTGCTAGGGGCTATGT	2000 bp
Mt-2F	AATAACAGATGGGGTAAGTCGTAACA	Mt-2R	GTGAAGAAGGCTACAGCAATTAAGAT	2000 bp
Mt-3F	GAGTTCAGACCGGAGC AATCCA	Mt-3R	CAGTTCCTGCGCCTGTTTCAAT	3500 bp
Mt-4F	CTACATGGTTTGATAAGAAGGGGAGT	Mt-4R	ATTGGTGATATTGCGTCTTGAAATCC	2000 bp
Mt-5F	CACTACACCAAACCTGAACCAAAGTA	Mt-5R	GATTGTGAATGGTGCTTCGTAGTATTC	2500 bp
Mt-6F	CACACAACTATCAATGAACATAGCAC	Mt-6R	TGAACTGAAATTGAATGATTGGAAGT	1500 bp
Mt-7F	AGTCTATGGCTCCACATTCTTCGT	Mt-7R	TAGGTTCCAGCATTTAGTCGTTCT	1500 bp
Mt-8F	AGAACCCCTATCACGAAAACGAAC	Mt-8R	GCTATTTTTACGGCGGTTTTTGGT	1500 bp
Mt-9F	AATCTCCTTATAAACCGAGAAGGT	Mt-9R	AGATTTAGTTCGTGGTTTGGCT	1500 bp
Mt-10F	ATCATTGCAGGACTACTAATCTCATCA	Mt-10R	ATTTCATCAGATGGAGATGTTAGATGGA	2000 bp
Mt-11F	GTCAACGCCACAGAATAAGC	Mt-11R	ATTCCGGTTTTGGGGATCGG	1000 bp
Mt-12F	GCCCTATCACCCAAACACTATTCT	Mt-12R	CAGTTTCATTGAGTTGGCAGACAT	1500 bp
Mt-13F	AACCCTTGTTAGTAAGATAC	Mt-13R	CGTTGTTATTGTTGCTTTGG	1500 bp
Mt-14F	TCCATTGACAGTTGGCGTAC	Mt-14R	CTATAACTAAGTCAAGCTTATGC	1500 bp
Mt-15F	ACCAATCTCAAACATAATTG	Mt-15R	GAGATTTACCAACCCTGAATG	2200 bp
Mt-16F	CAGAGCCAGGTAATCAATGC	Mt-16R	CAACTATACCTGCTCAGGCAC	2700 bp
Mt-17F	TCTGAGAAGCATTCTCATCA	Mt-17R	GTAAACTAATAGTTTCGATG	1500 bp
Mt-18F	GAACCACAACCTCTTGGTGC	Mt-18R	GGTAAGAAGGAGTATGGTGATTG	1800 bp
Mt-19F	ACCAATCTCAAACATAATCG	Mt-19R	CTGGCACGAGATTTACCAAC	1500 bp

**Table 2:** Gene length, start codon, and stop codon usage of mitochondrial genomes of four *P. sinensis* strains.

Mitochondrial Elements	Qingxi Huabie (HB) Strain				Jiangxi (JB) Strain				Japanese (RB) Strain				Qingxi Wubie (WB) Strain			
	Length (bp)	Intergenic Nucleotides (bp) *	Start Codon	Stop Codon	Length (bp)	Intergenic Nucleotides (bp) *	Start Codon	Stop Codon	Length (bp)	Intergenic Nucleotides (bp) *	Start Codon	Stop Codon	Length (bp)	Intergenic Nucleotides (bp) *	Start Codon	Stop Codon
tRNA^Phe^	69	0			70	0			70	0			69	0		
12SrRNA	979	0			979	0			980	0			979	0		
tRNA^Val^	70	0			70	0			70	0			70	0		
16SrRNA	1602	0			1604	0			1605	0			1603	0		
tRNA^Leu^	77	0			77	0			77	0			77	0		
NAD1	971	0	ATG	TAG	971	0	ATG	TAG	971	0	ATG	TAG	971	0	ATG	TAG
tRNA^Ile^	70	−1			70	−1			70	−1			70	−1		
tRNA^Gln^	71	9			71	9			71	9			71	9		
tRNA^Met^	69	0			69	0			69	0			69	0		
NAD2	1039	0	ATG	TAG	1039	0	ATG	TAG	1039	0	ATG	TAG	1039	0	ATG	TAG
tRNA^Trp^	73	11			73	11			73	11			73	11		
tRNA^Ala^	69	1			69	1			69	1			69	1		
tRNA^Asn^	74	−1			74	−1			74	−1			74	−1		
OL	34	−2			34	−2			34	−2			34	−2		
tRNA^Cys^	65	0			65	0			65	0			65	0		
tRNA^Tyr^	66	1			66	1			66	1			66	1		
COX1	1545	−5	GTG	AGA	1545	−5	GTG	AGA	1545	−5	GTG	AGA	1545	−5	GTG	AGA
tRNA^Ser^	71	1			71	1			71	1			71	1		
tRNA^Asp^	69	0			69	0			69	0			69	0		
COX2	687	1	ATG	TAA	687	1	ATG	TAA	687	1	ATG	TAA	687	1	ATG	TAA
tRNA^Lys^	73	1			73	1			73	1			73	1		
ATP8	165	−10	ATG	TAA	165	−10	ATG	TAA	165	−10	ATG	TAA	165	−10	ATG	TAA
ATP6	683	0	ATG	TAA	683	0	ATG	TAA	683	0	ATG	TAA	683	0	ATG	TAA
COX3	784	0	ATG	T	784	0	ATG	T	784	0	ATG	T	784	0	ATG	T
tRNA^Gly^	70	0			70	0			70	0			70	0		
NAD3	352	−2	ATG	TAG	350	0	ATG	T	350	0	ATG	T	350	0	ATG	TAG
tRNA^Arg^	70	0			70	0			71	0			70	0		
NAD4L	297	−7	ATG	TAA	297	−7	ATG	TAA	297	−7	ATG	TAA	297	−7	ATG	TAA
NAD4	1381	0	ATG	T	1381	0	ATG	T	1381	0	ATG	T	1381	0	ATG	T
tRNA^His^	70	0			70	0			70	0			70	0		
tRNA^Ser^	62	−1			62	−1			62	−1			62	−1		
tRNA^Leu^	74	0			74	0			74	0			74	0		
NAD5	1779	−5	ATG	TAA	1779	−5	ATG	TAA	1779	−5	ATG	TAA	1779	−5	ATG	TAA
NAD6	525	0	ATG	AGG	525	0	ATG	AGG	525	0	ATG	AGG	525	0	ATG	AGG
tRNA^Glu^	68	3			68	3			68	3			68	3		
Cytb	1140	3	ATG	TAA	1140	3	ATG	TAA	1140	3	ATG	TAA	1140	3	ATG	TAA
tRNA^Thr^	74	14			74	14			74	13			74	14		
tRNA^Pro^	71	0			71	0			71	0			71	0		
D-loop	1597				1660				1711				1699			

* Negative intergenic nucleotides indicate overlapping nucleotides between adjacent elements.

**Table 3:** Base composition statistics of complete mitochondrial genome and each element.

Mitochondrial Elements	Average Base Composition (%)						Average AT-Skew	Average GC-Skew
	T (U)	C	A	G	A + T	G + C		
tRNAs	28.21	21.67	34.90	15.22	63.11	36.89	0.11	−0.17
rRNAs	22.16	22.45	39.24	16.16	61.40	38.61	0.28	−0.16
D-loop	31.78	25.70	32.54	9.99	64.32	35.69	0.01	−0.44
ATP6	30.34	24.71	36.37	8.59	66.71	33.30	0.09	−0.48
ATP8	29.55	25.00	40.76	4.70	70.31	29.7	0.16	−0.68
COX1	30.91	23.53	29.92	15.65	60.83	39.18	−0.02	−0.20
COX2	27.62	23.94	36.64	11.79	64.26	35.73	0.14	−0.34
COX3	27.01	26.05	32.27	14.67	59.28	40.72	0.09	−0.28
Cytb	27.43	28.57	33.22	10.77	60.65	39.34	0.10	−0.45
NAD1	31.48	25.62	31.97	10.93	63.45	36.55	0.01	−0.40
NAD2	25.07	26.51	40.71	7.71	65.78	34.22	0.24	−0.55
NAD3	31.79	25.71	33.21	9.29	65.00	35.00	0.02	−0.47
NAD4	27.75	26.90	36.44	8.91	64.19	35.81	0.14	−0.50
NAD4L	30.89	26.43	34.01	8.67	64.90	35.10	0.05	−0.51
NAD5	26.25	29.06	35.26	9.43	61.51	38.49	0.15	−0.51
NAD6	13.29	31.28	48.50	6.95	61.79	38.23	0.57	−0.64
PCGs	27.64	26.41	36.10	9.85	63.74	36.26	0.13	−0.46
Complete genome	27.23	25.45	35.56	11.75	62.79	37.20	0.13	−0.37

**Table 4:** Comparison of variation loci in mitochondrial genomes of four *P. sinensis* strains.

Mitochondrial Elements	Total Number of Sites	Invariable Sites	Variable Sites	Singleton Variable Sites	Parsimony Informative Sites	Percentage of Variable Sites
Complete genome	17,064	16,581	483	436	47	2.83%
tRNAs	1541	1516	25	22	3	1.62%
rRNAs	2580	2536	44	44	0	1.71%
D-loop	1546	1433	113	74	39	7.31%
ATP6	684	668	16	16	0	2.34%
ATP8	165	159	6	6	0	3.64%
COX1	1545	1512	33	32	1	2.14%
COX2	687	675	12	12	0	1.75%
COX3	784	766	18	17	1	2.30%
Cytb	1140	1113	27	25	2	2.37%
NAD1	972	947	25	25	0	2.57%
NAD2	1041	1013	28	26	2	2.69%
NAD3	350	339	11	11	0	3.14%
NAD4	1381	1340	41	41	0	2.97%
NAD4L	297	286	11	11	0	3.70%
NAD5	1779	1734	45	45	0	2.53%
NAD6	525	507	18	18	0	3.43%

## Data Availability

The sequencing data of mitochondrial genomes involved in this study were deposited in GenBank under the accession numbers: OQ236104, OQ236105, OQ236106, and OQ236107.

## Supplementary Materials

The following supporting information can be downloaded at: https://www.mdpi.com/article/10.3390/biology12030406/s1, Table S1: Best partitioning scheme and model selected by PartitionFinder for phylogenetic analyses.
